# Improving Depression Severity Prediction from Passive Sensing: Symptom-Profiling Approach

**DOI:** 10.3390/s23218866

**Published:** 2023-10-31

**Authors:** Sabinakhon Akbarova, Myeongji Im, Suhyun Kim, Kobiljon Toshnazarov, Kyong-Mee Chung, Junghyun Chun, Youngtae Noh, Young-Ah Kim

**Affiliations:** 1 Research and Development Department, Huno, Seoul 04146, Republic of Korea; sabinakhon.akbarova@gmail.com (S.A.); ymj@huno.kr (M.I.); ksh@huno.kr (S.K.); oz@huno.kr (J.C.); 2 Energy AI, KENTECH, Naju 58330, Republic of Korea; qobiljon@kentech.ac.kr; 3 Department of Psychology, Yonsei University, Seoul 03722, Republic of Korea; kmchung@yonsei.ac.kr

**Keywords:** digital phenotyping, smartphone sensing, depressive symptoms, machine learning, EMA

## Abstract

Depression is a significant mental health issue that profoundly impacts people’s lives. Diagnosing depression often involves interviews with mental health professionals and surveys, which can become cumbersome when administered continuously. Digital phenotyping offers an innovative approach for detecting and monitoring depression without requiring active user involvement. This study contributes to the detection of depression severity and depressive symptoms using mobile devices. Our proposed approach aims to distinguish between different patterns of depression and improve prediction accuracy. We conducted an experiment involving 381 participants over a period of at least three months, during which we collected comprehensive passive sensor data and Patient Health Questionnaire (PHQ-9) self-reports. To enhance the accuracy of predicting depression severity levels (classified as none/mild, moderate, or severe), we introduce a novel approach called symptom profiling. The symptom profile vector represents nine depressive symptoms and indicates both the probability of each symptom being present and its significance for an individual. We evaluated the effectiveness of the symptom-profiling method by comparing the $F_{1}$ score achieved using sensor data features as inputs to machine learning models with the $F_{1}$ score obtained using the symptom profile vectors as inputs. Our findings demonstrate that symptom profiling improves the $F_{1}$ score by up to 0.09, with an average improvement of 0.05, resulting in a depression severity prediction with an $F_{1}$ score as high as 0.86.

## 1. Introduction

Depression, as defined by the World Health Organization (WHO), is a clinically identifiable syndrome characterized by observed behavioral patterns and experienced symptoms that influence personal functioning [1]. It is a prevalent mental health disorder affecting approximately 4.7% of the global population within any taken 12-month period [2]. Tragically, depression is closely associated with suicide, making it the most frequently reported psychiatric disorder among those who commit such acts [3]. The recent COVID-19 pandemic has further underscored the urgency of addressing depression prevention, recognition, and treatment on a global scale due to its adverse impact on the mental well-being of millions [4]. Nevertheless, diagnosing depression is challenging due to the absence of diagnostic laboratory tests and the interpersonal variations in experienced symptoms. Mental health professionals commonly rely on interviews and survey instruments for diagnosis, which can be burdensome and impractical for continuous use.

The widespread use of smartphones offers new opportunities for screening, diagnosing, assessing, and monitoring individuals with depression by capturing various dimensions of human behavior. This field, known as digital phenotyping, entails quantifying an individual’s phenotype in real-life situations using data from personal digital devices [5]. Passive sensing, in particular, allows short-term detection of depressive symptoms without requiring active input from users, thereby potentially mitigating long-term negative consequences [6]. A growing body of research supports the efficacy of passive smartphone sensor data in distinguishing individuals with depressive disorder from controls (e.g., [6,7,8,9,10,11]).

Existing studies on depression detection using smartphone data primarily focus on binary classification, distinguishing between individuals who are depressed and those who are not [12]. However, it is important to note that the Diagnostic and Statistical Manual of Mental Disorders (DSM-5) [13], the psychiatric diagnostic system, lists nine symptoms used to diagnose major depressive disorder, and the number of symptoms varies from person to person. In some cases, individuals may be diagnosed with depression without experiencing any shared symptoms [14]. Therefore, it is crucial to consider the nature of specific depressive symptoms and signs, as they may manifest in diverse clinical phenotypes [15].

In this study, our aim is to explore the feasibility of automatic depression detection that encompasses both the severity of depression (none/mild, moderate, severe) and the specific depressive symptoms as assessed by the Patient Health Questionnaire-9 (PHQ-9) [16]. We collected data from 381 participants over a period of three months or more, utilizing 19 sensors for Android and 11 sensors for the iOS operating system. The set of sensor information collected from smartphones covers multiple dimensions of behavioral data, including physical activity, social activity, mobility, and device usage. The sensor data are complemented by in situ Ecological Momentary Assessments (EMAs) [17]. We employ machine learning to detect the presence or absence of specific depressive symptoms and predict three levels of depression severity. Furthermore, to enhance the accuracy of depression severity prediction beyond the direct utilization of sensor data as input for machine learning models, we introduce a novel approach involving symptom profiling. A symptom profile vector is a multi-dimensional data representation consisting of nine elements, with each element corresponding to one of the depressive symptoms outlined in the PHQ-9 questionnaire. This concept arises from a comprehensive understanding of which depressive symptoms are present and how indicative they are of depression for an individual. The main contributions of our study include the following:

- We achieve accurate predictions of both behavioral and cognitive depressive symptoms with $F_{1}$ scores reaching up to 0.83 for Android and 0.76 for iOS datasets by utilizing a wide range of sensor data.
- We introduce a novel method for predicting depression severity, which utilizes the symptom profile vector. This approach enhances the $F_{1}$ score by up to 0.09 (0.05 on average). The improvement is consistent across both Android and iOS datasets and is observed across all tested machine learning models.
- We investigate characteristics of the symptom profile vector among three depression severity groups. It proves to be a viable representation of PHQ-9 self-reports, particularly for the non-depressed and mildly depressed groups, distinguishing among the three levels of depression severity.

The rest of the paper is organized as follows. We begin by providing a brief description of the related work in Section 2. Subsequently, Section 3 outlines the data-collection process, covering the study procedure and the data-collection system. In Section 4, we present a comprehensive overview of the data-processing procedures applied to both the passively collected data and the self-reports. The concept and calculation of the symptom profile vector are detailed in Section 5. Presenting the findings, Section 6 showcases the results obtained from our study. The limitations of the current study are discussed in Section 7. Lastly, Section 8 concludes the paper.

## 2. Related Work

We focus on previous research that investigates the relationship between smartphone sensor data and depression in non-clinical cohorts. Additionally, we examine approaches to predicting depressive symptoms and severity. We summarize the previous studies in Table 1, including information on the number and type of participants, duration of data collection, questionnaires used for EMAs, assessments for pre-/post-tests, and devices utilized for collecting the sensor data.

Recent studies have used digital phenotyping to predict mental well-being and study the association between passively collected data and depression [7,8,18,19,20,21,23,24,25,26]. Wang et al. [18] conducted a study to evaluate mental health, including depression, stress, sleep, activity level, mood, sociability, and academic performance, in undergraduate and graduate students. Notably, the depression severity exhibited a statistically significant correlation with sleep duration measured via an accelerometer, microphone, and light sensor, conversation frequency and duration measured via a microphone, and the number of Bluetooth encounters. Similarly, Ben-Zeev et al. [19] examined the association between smartphone sensor data and depression, stress, and subjective loneliness. Their investigation indicated that changes in depression levels were associated with sensor-measured speech duration, geospatial activity, and sleep duration. Canzian et al. [7] analyzed mobility trace data for predicting depressive mood. In the general model, the authors performed binary classification and achieved sensitivity and specificity values of 0.74 and 0.78, respectively. In the personalized models, the average sensitivity and specificity values were 0.71 and 0.87, respectively. In a study by Saeb et al. [8], participants with depressive symptoms exhibited a significant difference from their non-depressed counterparts in terms of normalized entropy, location variance, circadian movement, and duration and frequency of phone usage. The study yielded an accuracy of 86.5% for binary depression classification, using the normalized entropy feature. In a subsequent replication study, Saeb et al. [20] extended their previous work on the relationship between depressive severity and geographic location using the StudentLife dataset [18]. Their findings indicated that GPS features are useful in predicting depression severity 10 weeks before the assessment, thereby offering an early warning for potential depression. Boukhechba et al. [21] conducted a study to monitor depressive symptoms and social anxiety in college students using sensor data. They found that mobility patterns measured via GPS (time spent at service places, time spent outside the city, time spent at someone else’s home) and activity level measured via an accelerometer sensor were correlated with the level of depression measured via the Depression, Anxiety and Stress Scale (DASS) [27]. Xu et al. [23] proposed a novel approach for capturing contextual behaviour features, referred to as contextually filtered features, that utilizes associate rule mining. This technique was found to improve the accuracy of depression prediction by an average of 9.7% when compared to using unimodal features alone. Razavi et al. [24] investigated the relationship between depressive symptoms and smartphone-usage behaviour, including the number of saved contacts, SMS usage, and duration and frequency of calls. The random forest classifier model for classifying depression showed out-of-sample balanced accuracy of 76.8%. The accuracy improved to 81.1% when incorporating demographic information such as age and gender. In their study, Opoku Asare et al. [25] found that screen data and Internet connectivity had the most significant impact on predicting binary depressive state (depressed or non-depressed). The classification model achieved an $F_{1}$ score of 0.88 to 0.94. Ross et al. [26] introduced an innovative method for predicting fluctuations in depression severity, focusing on clustering accelerometer data specifically during participants’ typing activities. The model achieved an accuracy of around 95%, accompanied by an area under the ROC curve of 97%.

Several studies have investigated the use of sensor data to predict and monitor depression. However, fewer studies have focused on specific depressive symptoms and severity using sensor data. Wang et al. [22] conducted a study to investigate the correlation between depression severity, measured via PHQ-8 and PHQ-4, and sensor data representing depressive symptoms based on DSM-5 criteria. They developed a model using the sensor data to predict whether the student was depressed every week and achieved an accuracy of 69.1%. Narziev et al. [6] aimed to predict depression levels (normal, mild, moderate, severe) based on five depression indicators derived from passive sensor data, including physical activity, mood, social activity, sleep, and food intake. The study achieved an average True Positive Rate of 0.96. Ware et al. [12] explored the feasibility of predicting both behavioral and cognitive depressive symptoms from GPS data collected from smartphones and the institution’s WiFi infrastructure and achieved an $F_{1}$ score of up to 0.86 for the sleep-related symptom.

## 3. Data Collection

This section begins with a description of the participant-recruitment process and the data-collection system, which includes the client application, cloud platform, and web server. The section further elaborates on the privacy measures that were taken to safeguard the collected data, as well as the data quality monitoring functionality that we employed.

### 3.1. Study Procedure

To recruit participants for this study, we distributed a recruitment statement through various channels, including social networking sites (Facebook, Instagram), multiple universities, and online communities, as well as poster advertisements in subways. A total of 2620 individuals visited the research information homepage, of which 2080 proceeded to undergo a screening process. The screening process involved checking participants’ smartphone availability, psychiatric history, and PHQ-9 scores. Only those individuals who met the predetermined inclusion criteria were invited to review research guide videos, complete research consent forms, and install the designated research application on their devices. The guide videos provided participants with information about the data-collection procedures and instructions for responding to EMAs. The duration of data collection for this study was predetermined as a fixed period of three months, with an option for participants to extend their participation for additional three months. The duration of data collection was determined in accordance with [28], who reported that the duration of depressive episodes may vary; however, the median duration of a depressive episode is typically three months.

Ultimately, 704 participants installed the research application. We provided the recruited participants with a comprehensive research consent form and a detailed description of the study, including its purpose, methods of participation, potential risks and benefits. This study strictly adhered to ethical guidelines established by the Yonsei University Institutional Review Board (IRB) and received approval under IRB number 7001988-202108-HR-1101-08.

### 3.2. Data-Collection System

**Figure 1:**
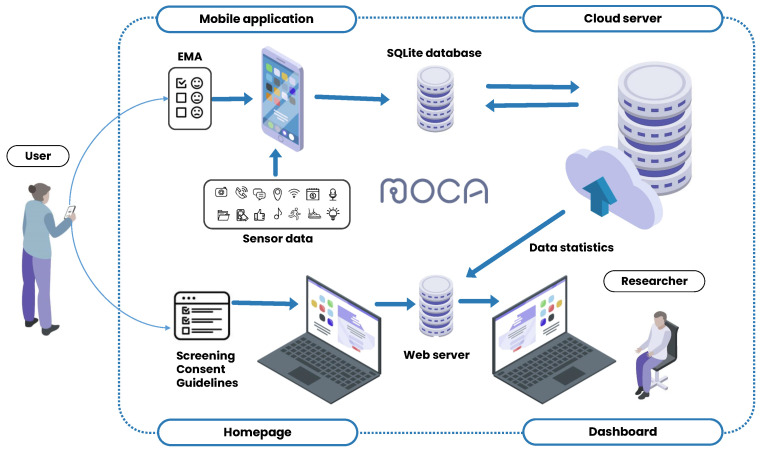
Data-collection system components.

As shown in Figure 1, the sensing system in this study was composed of three primary components: the client application, the cloud data-collection platform, and a web server.

**Figure 2:**
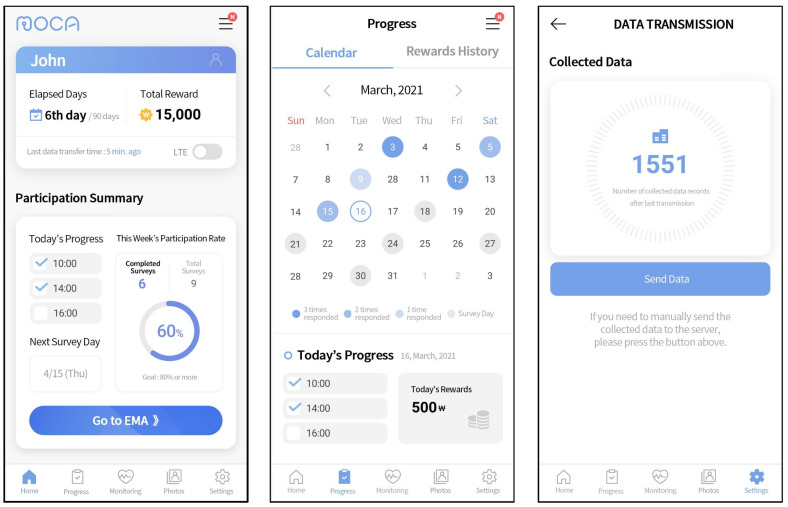
User interfaces of MOCA application (dashboard, progress calendar, and manual data transmission).

The client application, MOCA, used in this study, was made available free of charge through the research website. We developed the application for both Android and iOS operating systems. MOCA served two main functions: the continuous passive collection of sensor data and the delivery of in situ EMAs [17]. Figure 2 illustrates sample screenshots from the Android version of the application. The initial screenshot shows the application’s home screen, where users monitored the number of days elapsed since the start of data collection, the most recent data transmission to the server, and the daily and weekly progress of EMA responses. The subsequent screenshot portrays the calendar interface, facilitating the review of the upcoming EMA schedule and response history. The last screenshot demonstrates the data-uploading feature, which allowed users to manually send data to the server by pressing a button in cases where automatic transfer encountered issues. MOCA collected sensor data passively in the background without requiring any actions from participants. In this study, we collected a total of 19 passive data sources for Android and 11 for iOS. A detailed description of the data collected from each source, along with their collection intervals for Android and iOS, is provided in Table 2. The data sources and collection intervals vary between Android and iOS applications due to differences in their operating system platforms. Therefore, we analyzed the datasets separately for each platform. The data sources are categorized as either interval-based or event-based, depending on their collection intervals. Interval-based data sources are defined by the application for a specific collection period, whereas event-based data sources are triggered by specific events such as phone unlocking, changes in physical activity, or application opening. Additionally, Table 2 shows which of the data sources were optional for collection, meaning that participants could choose to provide or withhold certain data based on their preferences. The optional data sources included social networking service, stored media, microphone, and camera.

In the context of EMA, we collected the PHQ-9, a validated nine-item depression module derived from the full PHQ, as a self-report measure, owing to its widespread acceptance as a screening tool for depression [16]. The PHQ-9 evaluates both behavioral and cognitive depressive symptoms, such as depressed mood, lack of interest, sleep problems, fatigue, appetite problems, feelings of worthlessness, concentration problems, psychomotor retardation, and suicidal ideation. Moreover, we included a reliability check question, consisting of a Likert-type paraphrase of a PHQ-9 item that assessed the symptom of depressed mood. The client application incorporated several features to facilitate self-report collection. Firstly, we implemented a randomized questioning sequence to minimize the potential for automatic responses. Secondly, the application recorded the EMA response time duration for further EMA filtering described in Section 4.1. Thirdly, users were provided with a one-hour window to submit their response following an EMA push notification. Participants who had not yet submitted their answers, received a push notification reminder 15 min before the response deadline. Finally, the application offered two possible EMA time schedules (10 a.m., 2 p.m., 6 p.m., or 12 p.m., 4 p.m., 8 p.m.) to enable participants to customize their EMA reception time based on their daily routine. The decision to administer EMA questionnaires within a specified time frame was informed by the findings of Narziev et al. [6], who demonstrated that optimal EMA response rates were between 10 a.m. and 10 p.m. To mitigate the burden on participants from frequent survey responses, we implemented a schedule of administering EMAs once every three days, with a two-day interval between each administration.

The second component of the sensing system was the EasyTrack cloud platform [29]. It offered a robust and efficient means for data collection by utilizing the Django [30] framework and the Apache Cassandra NoSQL database [31]. The platform’s main functions included receiving and transmitting data between the client application and the platform, as well as monitoring the quality of the collected data. The collected sensor data were aggregated into small data packets by the client application and transmitted to the cloud server via WiFi or LTE connection, according to user preferences.

The last component of the sensing system was a dedicated web server for participant management. This server comprised a homepage and a dashboard. The homepage incorporated various functions, such as research participation application and consent, screening results, and personalized progress guidance for each participant. The dashboard was equipped with features to seamlessly monitor participants’ progress and manage the collected research data, integrating information from both the homepage and the client application.

### 3.3. Privacy Considerations

We implemented measures to safeguard the collected sensor data and self-assessments, given the sensitive nature of the information. Regarding the data themselves, we did not collect any content of conversations or messages; the sensor data comprised numeric information such as noise levels and the count of phone calls. The mobile application securely transmitted the collected data to the cloud server using the SSL protocol through a TCP connection. To ensure anonymity, each participant was assigned a randomly generated ID number by the cloud server. Access to the cloud platform and web server was restricted to authorized personnel. The administrator had the sole authority to invite research personnel to access the server via their email accounts. These security measures were implemented to protect participants’ privacy and maintain the confidentiality of their data.

### 3.4. Data Quality Monitoring

The data-collection system incorporated specialized functionality aimed at enhancing data completeness. Firstly, the client application monitored the frequency of participant responses to EMA surveys, specifically focusing on identifying those with a response rate below 80%. This detection process occured weekly, and mobile notifications were sent to users to encourage more frequent participation. Secondly, the application closely monitored data transmission to the server, particularly in situations where automated data transmission encountered issues. Participants received push notifications if data had not been transmitted for 72 h or longer. The application integrated a manual data-transmission function, which allowed participants to directly send data to the server, thereby ensuring that no valuable data were lost.

## 4. Data Processing

In this section, we discuss a comprehensive set of procedures that we applied to preprocess data and transform them into a suitable format for further analysis. We first describe the preprocessing of the survey data, followed by the methods utilized for sensor data.

### 4.1. EMA Data

We utilized two filters to enhance the reliability of the collected survey data. Firstly, we excluded EMAs where the reliability check question was answered incorrectly. Secondly, we removed EMAs that were answered too quickly. Among all the questionnaires administered, 3% displayed a discrepancy of greater than one point between the responses to the PHQ-9’s second item (“Feeling down, depressed or hopeless”) and the corresponding trap question (a restated version of the item), rendering them invalid. For each of the remaining EMAs, we computed the Speeder Index as a criterion for filtering responses based on their response duration. We followed the methodology proposed by Canzian et al. [7] and set the threshold for voiding an EMA to 10% of the lowest Speeder Index. The Speeder Index calculation starts with computing the median EMA response time for all participants. For questionnaires with a completion time equal to or longer than the calculated median, the Speeder Index equals 1. For the remaining self-reports, the Speeder Index is the ratio between the EMA response time and the median response time.

To determine the daily PHQ-9 score, we applied different methods depending on the availability of the self-report on the given day. For the days when a participant completed the assessment at least once, the daily PHQ-9 score was calculated as an average of the reported self-assessments. For days when no EMAs were reported, we employed linear interpolation by following the current practices in the area when dealing with missing EMA values [23]. We applied the linear interpolation for each item score individually for each participant. The PHQ-9 score for the interpolated days was obtained by summing the individual item scores. The total PHQ-9 score was classified into three distinct categories representing different levels of depression severity: none/mild, moderate, and severe. The PHQ-9 score served as a measure of severity, ranging from 0 to 27, as each of the 9 items was scored from 0 to 3. According to [16], specific cut-off scores represent the severity levels: 5, 10, 15, and 20, which denote mild, moderate, moderately severe, and severe depression, respectively. In our study, the three cohorts were categorized as follows: the normal and mild groups (PHQ-9 scores below 10), the moderate and moderately severe groups (PHQ-9 scores between 10 and 19), and the severe group (PHQ-9 scores 20 and more). For symptom scores, we adopted the approach presented by Ware et al. [12], who considered scores greater than 0 on the Likert scale as indicative of symptom presence. Due to the limited number of samples with scores 2 and 3, we grouped scores of 1, 2, and 3. As a result, our classification of depressive symptoms (Section 6.1) consisted of two classes: the presence or absence of each symptom.

### 4.2. Sensor Data

This section provides a description of the methods employed to preprocess sensor data and generate machine learning-ready features. In the first subsection, we outline the exclusion criteria for data sources. The subsequent subsection elaborates on the raw data preprocessing phase, which encompasses the cleaning of noisy data records, imputation of missing data, and data transformations. Following the raw data preprocessing phase, we proceed with feature extraction.

#### 4.2.1. Data Sources Exclusion

As described in Section 3.2, our data comprised data sources from two platforms: Android (19 sources) and iOS (11 sources). However, due to various data quality issues discovered during the primary Exploratory Data Analysis (EDA), some of the collected data sources were not used for data analysis. Firstly, we excluded data sources with low or no variability in measurements on an individual level, namely significant motion (Android) and calendar (Android, iOS). Another criterion for excluding data sources was the absence of data from multiple participants. We omitted the analysis of Social Networking Service (SNS), Music, and Short Message Service (SMS) data. The collection of SNS data was voluntary, as participants needed to log into their social media accounts via our application to provide this information. Consequently, only 17% of participants provided us with social media data. Regarding music data, MOCA captured song information from the most widely used music players in Korea, including Melon [32], Genie [33], Flo [34], and others. However, almost 30% of participants lacked music data, which might be attributed to the utilization of less common applications. Prior research [35] states that SMS data serve as an indicator of social interaction. However, in Korea, the predominant method of messaging is an application called KakaoTalk [36]. Therefore, the frequency of KakaoTalk usage was employed as an indicator of social interaction. Regarding the camera sensor, our intention was to utilize it for capturing face photos and subsequently apply facial emotion recognition. However, the COVID-19 pandemic necessitated the use of face masks by a majority of users, rendering the analysis of emotions from the captured photos challenging.

#### 4.2.2. Data Preprocessing

The initial stage involved the identification and removal of noisy data records, specifically out-of-range values. These measurements, which deviated from the anticipated range, arose due to data-acquisition or data-entry errors. The expected range of values was established for each data source individually, taking into account its unique characteristics. For example, for data sources that captured time duration, such as activity recognition, calls, and others, the expected range of values was constrained to positive values since duration cannot be negative. To cleanse GPS data of erroneous measurements or outliers, we leveraged a widely employed clustering algorithm, DBSCAN [37]. This density-based algorithm is effective in identifying low-density regions as outliers. The algorithm requires two parameters, namely epsilon (maximum distance between points) and cluster size (minimum number of points required to form a cluster). We adopted the recommended parameter settings from [9], wherein epsilon was set to 0.0005 and the minimum duration of stay at a cluster was set to 2.5 h, which allowed us to calculate the corresponding number of points required to form a cluster.

Following the erroneous data record removal, we proceeded with the implementation of missing data imputation techniques. The absence of data measurements could be caused by diverse factors, including but not limited to application errors resulting in untriggered events for event-based data sources. Additionally, missing data might arise due to the termination of data-acquisition services by the device operating system, or the non-existence of data records. Furthermore, data omissions might originate from data-entry discrepancies during client-side database input and its subsequent transmission to the server-side database. To address this, we utilized the linear interpolation method for imputing missing data for interval-based data sources. However, we refrained from applying the missing data imputation method to event-based data sources due to the challenge of distinguishing between missing and non-existent data in such cases.

As a final step of data preprocessing, we applied a Box–Cox transformation [38] individually to each participant’s data. The Box–Cox transformation is a widely recognized data-transformation technique that is employed to enhance the reliability and validity of analyses involving continuous data that do not follow a normal distribution or display heteroscedasticity. The Box–Cox transformation involves a rescaling and unit alteration of the measurement values, rendering it an appropriate choice for our numerical data. However, it should be noted that this transformation was not suitable for latitude and longitude values as we required the original GPS values for further feature extraction.

#### 4.2.3. Feature Extraction

The data analysis incorporated two types of features: demographic information about the participants and features extracted from sensor data. In terms of demographics, we utilized gender and age, as this information improves the accuracy of predicting depression based on previous research [24]. For the age variable, we transformed the age into three age groups: below 30 years, between 31 and 45 years, and 46 years or more.

Regarding the sensor data, we categorized data sources into three categories based on their specific measurement characteristics: duration-based, value-based, and quantity-based. It should be noted that certain data sources belonged to multiple categories. For instance, keystroke log data, which recorded typing characteristics, was analysed for both quantity-based information (such as the number of key presses) and duration-based information (such as typing duration). Hence, the particular features derived from the raw data rely on the mentioned categorizations:

- Duration-based data sources measured the amount of time for a particular activity such as walking or talking over the phone. We calculated the mean and variance of activity durations over a specified period of time.
- Value-based data sources measured specific characteristics such as acceleration or sound energy. Analogous to duration-based data sources, the features extracted from these sources were determined by computing the mean and variance of measurements captured over a designated time interval. While GPS sensors were classified as value-based data sources, the approach used for computing their features differed from that of other value-based data sources. Specifically, the computation of location features was predicated on a methodology described in [8].
- Quantity-based data sources measured the number of occurrences of a particular action or the quantity of something. A pedometer sensor recording the number of steps taken represented an example of a quantity-based data source. The cumulative count of occurrences within a specified time frame was computed for this class of data source.

As described in Section 4.1, self-reports were aggregated on a daily basis. Consequently, we calculated features over 24 h intervals using preprocessed sensor data. Table 3 contains an overview of analyzed data sources and the corresponding extracted features from each data source. Additionally, the table includes device operating system information, because not all the listed data sources are available for both platforms.

After performing feature extraction, we obtained a total of 68 features for the Android platform and 34 features for iOS. To reduce the dimensionality of the dataset, we initially examined the extracted features for low variability, as these features are often uninformative and can have a detrimental effect on the performance of machine learning models. We used a threshold of 0.05 to identify quasi-constant features, which is a common practice. Subsequently, we evaluated the features based on the amount of missing data across all participants. As a rule of thumb, we employed a threshold of 20% to discard features. Following the feature removal based on low variability and missing data, Android had 43 remaining features, while iOS had 31.

## 5. Analysis Methods

In this section, we present our methodology designed to improve the accuracy of depression severity prediction. Our approach focuses on capturing a representation of the Diagnostic and Statistical Manual of Mental Disorders (DSM-5) [13] depressive symptoms, enabling the accurate prediction of depression severity. To achieve this, we introduce a novel metric called *symptom profile vector*, which is composed of nine elements. Each element of this vector corresponds to one of the depressive symptoms enumerated in the PHQ-9 questionnaire.

### 5.1. Symptom Profile Concept and Calculation

The key idea behind the concept of the symptom profile vector is the recognition of the depressive symptoms heterogeneity (which symptoms are present) and their significance (which symptoms are most indicative of depression). We consider symptom heterogeneity because it acknowledges the wide range of symptom variations among individuals with depression [40]. Individuals with similar PHQ-9 assessment sum scores can have different syndromes. Symptoms significance refers to variations in the interpretation and importance of similar symptoms among individuals. To exemplify the utility of the symptom significance, consider two hypothetical individuals, Person A and Person B. Both Person A and Person B are experiencing the same depressive symptom (e.g., problems with appetite and/or sleep). For Person A, the symptom is associated with lifestyle routine or some mental or physical health problems other than depression, while for Person B the depressive symptom was caused by depression. This example illustrates the diverse interpretations of symptoms and their significance for different individuals.

**Figure 3:**
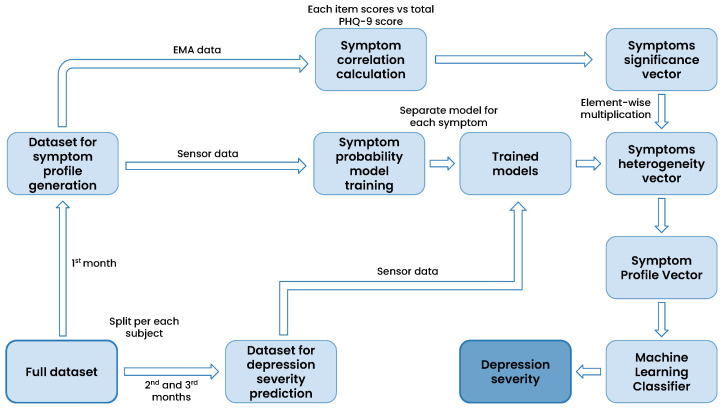
The high-level pipeline depicting the integration of symptom profile and machine learning for depression severity prediction.

Figure 3 presents the high-level pipeline employed in the proposed symptom-profiling approach. Initially, the dataset is split into two distinct subsets. This partitioning is carried out individually for each participant, where the first month’s accumulated data are designated for symptom profile vector generation, while the remaining data are reserved for depression severity prediction. The symptoms significance vector is derived from the collected self-reports within the first subset. This vector is constructed by calculating the absolute value of the Pearson correlation between item scores related to a particular symptom and the total PHQ-9 scores recorded during the first month of data collection. As a result, each participant is represented by a nine-element vector referred to as the symptom significance vector ($\vec{SS}$). Each element of the vector ranges from 0 to 1 and indicates the significance of the respective symptom.

Sensor data collected during the first month is used to train models tasked with predicting the likelihood of the presence of a depressive symptom. These probabilities are determined by utilizing the predict_proba() method of the XGBoost model [41]. Consequently, nine distinct models are trained, each associated with a specific depressive symptom. In the subsequent phase, the sensor data of the dataset for depression severity prediction (2nd and 3rd months of data) are fed into the probability-prediction models, yielding the likelihood of each symptom’s occurrence. The resulting vector is referred to as the symptoms heterogeneity vector ($\vec{SH}$). The vector comprises nine elements ranging from 0 to 1 and indicates the probability of respective depressive symptoms on a given day.

The symptom profile vector ($\vec{SP}$) is calculated through element-wise multiplication, also known as the Hadamard product, between the symptom significance vector $\vec{SS}$ and the symptoms heterogeneity vector $\vec{SH}$ (as shown in Equation (1)). This resulting symptom profile vector is subsequently utilized as input for a machine learning classifier, enabling the prediction of depression severity for the second and third months of data collection (Section 6.2).

(1) $$\vec{SP}=\vec{SH}⊙\vec{SS}$$

To illustrate the conceptual meaning of the symptom profile vector, we revisit the example mentioned earlier in this section. As a recap, Person A and Person B are experiencing the same depressive symptom. However, for Person A, the symptom is not caused by depression, in contrast to Person B. For both of them, the element of the symptom heterogeneity vector associated with the experienced symptom is expected to be close to 1, indicating a high probability of the symptom being present. However, the symptom significance vector element corresponding to the symptom is expected to be higher for Person B than for Person A. Consequently, when we multiply the elements of the symptom significance vector by the elements of the symptom heterogeneity vector, the value of the corresponding element in the resulting symptom profile vector will be lower for Person A than for Person B, reflecting the diminished influence of the symptom in predicting depression for Person A.

### 5.2. Weighted Symptom Profile

According to the diagnostic criteria outlined in the DSM-5 for depression, an individual must exhibit five or more symptoms to receive a depression diagnosis [13]. Notably, at least one of these symptoms must be either a depressed mood or a loss of interest or pleasure in activities. With the goal of improving the predictive accuracy of depression severity, we investigated the impact of artificially amplifying the significance of these two symptoms. To achieve this, we doubled the weight assigned to the elements within the symptom profile vector corresponding to depressed mood and anhedonia when using the machine learning classifier. In contrast, the previously described symptom-profiling method involved the machine learning classifier autonomously determining the most optimal feature weights for the algorithmic process. By explicitly increasing the importance of these two symptoms, our aim was to assess whether this adjustment yields superior prediction accuracy for depression severity and a more refined differentiation among depression levels. The results of experiments with the weighted symptom profile vector are shown at the end of Section 6.2.

## 6. Results

We considered data collected from July 2021 to June 2022 by participants who finished at least three months of data collection. A total of 381 participants (252 with Android devices and 129 with iOS devices) completed the study. As for the demographic characteristics of the participants, our dataset consisted of 63% female and 39% male participants aged between 18 and 61 years (mean age = 28, standard deviation = 9.37).

### 6.1. Predicting Depressive Symptoms

**Figure 4:**
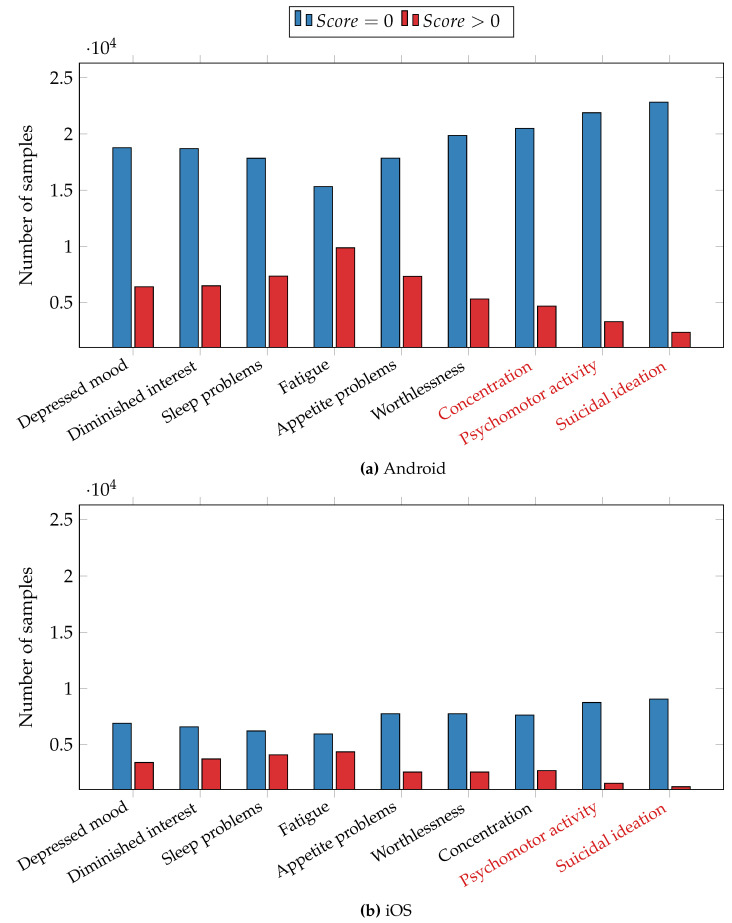
Distribution of samples for depressive symptoms. (**a**) The Android dataset and (**b**) the iOS dataset. Symptoms marked in red are excluded from the analysis.

As an initial step in our data analysis, we conducted machine learning tests to predict the presence or absence of depressive symptoms. To accomplish this, we trained separate XGBoost models, each corresponding to a specific depression symptom outlined in the PHQ-9 depressive questionnaire. The classification process was performed for daily self-reports, and the preprocessing procedure, including binarization, is detailed in Section 4.1. Figure 4 displays the histograms depicting the distribution of ground truth data. Symptom labels marked in red indicate significant imbalances in sample representation, with one class containing more than 4 times or less than 1/4 of the samples compared to the other class. These imbalanced symptoms were subsequently excluded from the analysis, following the approach outlined by Ware et al. [12]. To address the remaining data imbalance in symptoms, we employed the Synthetic Minority Over-sampling Technique (SMOTE) [42] on the training set. To prevent overfitting, we utilized the leave-one-subject-out (LOSO) cross-validation method, whereby no data from a given user were used for both training and testing. We evaluated the models’ predictive capabilities using $F_{1}$ score, precision, and recall, calculated with the scikit-learn library [43]. Precision quantifies the accuracy of identifying true positive cases, where a depressive symptom is present, among all cases predicted as positive. Recall assesses the model’s ability to identify all true positive cases among all actual positive cases. The $F_{1}$ score, a harmonic mean of precision and recall, is defined as 2×(precision×recall)/(precision+recall).

Classification results are presented in Table 4, with Android and iOS results presented separately due to significant differences in data-collection mechanisms. For each depressive symptom, the Android dataset consisted of 25,176 samples, while the iOS dataset comprised 10,290 samples. As previously mentioned, certain symptoms were excluded from the analysis. Specifically, for the Android dataset, the excluded symptoms were inability to concentrate, psychomotor activity retardation or agitation, and suicidal ideation. For the iOS dataset, we excluded psychomotor activity change and suicidal thoughts symptoms.

We first discuss the results obtained from the Android dataset. All $F_{1}$ scores surpassed 0.77, except for the fatigue symptom, which had a score of 0.69. $F_{1}$ scores ranged from 0.69 to 0.83. Particularly noteworthy were the highest $F_{1}$ scores attained for the feeling of worthlessness symptom (0.83), the diminished interest and depressed mood symptoms (0.80). It is interesting to note that all three of these symptoms are cognitive, suggesting the possibility of monitoring cognitive symptoms through the analysis of digital behaviour data.

The lower section of Table 4 displays the results from the iOS dataset, with $F_{1}$ scores ranging from 0.63 to 0.76. The highest $F_{1}$ scores were observed for the inability to concentrate symptom (0.76), the feeling of worthlessness symptom (0.75), and the depressed mood symptom (0.70). In line with the findings from the Android dataset, symptoms with cognitive characteristics exhibited the highest predictive performance. However, the $F_{1}$ scores achieved for the iOS dataset are lower compared to those for the Android dataset. One potential explanation is the smaller number of data sources available for iOS in comparison to Android, coupled with a larger amount of missing data for iOS. The iOS operating system requires the application to be open in the app switcher to facilitate sensor data collection. When users swipe up to close the application, it is removed from the background, resulting in larger data gaps in the iOS dataset compared to the Android dataset.

### 6.2. Predicting Depression Severity

In this section, we present the findings regarding the classification of depression severity for the second and third months of data collection for each participant as described in Section 5. The classification involved three distinct classes, namely none/mild depression, moderate depression, and severe depression. The detailed process of converting total PHQ-9 scores into the three depression group categories is outlined in Section 4.1. We conducted a comparative analysis to evaluate the performance of machine learning models in two scenarios: (1) employing sensor data directly as input and (2) utilizing symptom profile vectors as input. To ensure the consistency of results, we tested our approach using four distinct models: XGBoost [41], LightGBM [44], CatBoost [45], and Support Vector Machine (SVM) [46]. We computed weighted averages of $F_{1}$ score, precision, and recall using the Python scikit-learn library as performance metrics. Weighted precision, recall, and $F_{1}$ score are commonly used in multiclass classification, especially when dealing with imbalanced data. This is because unweighted precision, recall, and $F_{1}$ score can be heavily influenced by the dominant class. The process for calculating these metrics in multiclass classification involves first computing them separately for each class, treating each class as the positive class. Then, we calculate weighted average scores, where each $F_{1}$ score, precision, and recall is multiplied by the number of samples in the corresponding class before averaging. To assess the generalizability of our model in predicting depression severity for unseen participants’ data and its ability to combat overfitting, we employed the cross-validation method used by Ware et al. [12]. Leave-one-user-out cross-validation evaluates the model’s generalization capability by repeatedly testing it on unseen participants’ data. Specifically, the leave-one-user-out cross-validation technique ensures that no data from one user are included in both the training and test sets, thereby evaluating the model’s capacity to learn data patterns rather than memorizing noise and overfitting.

**Figure 5:**
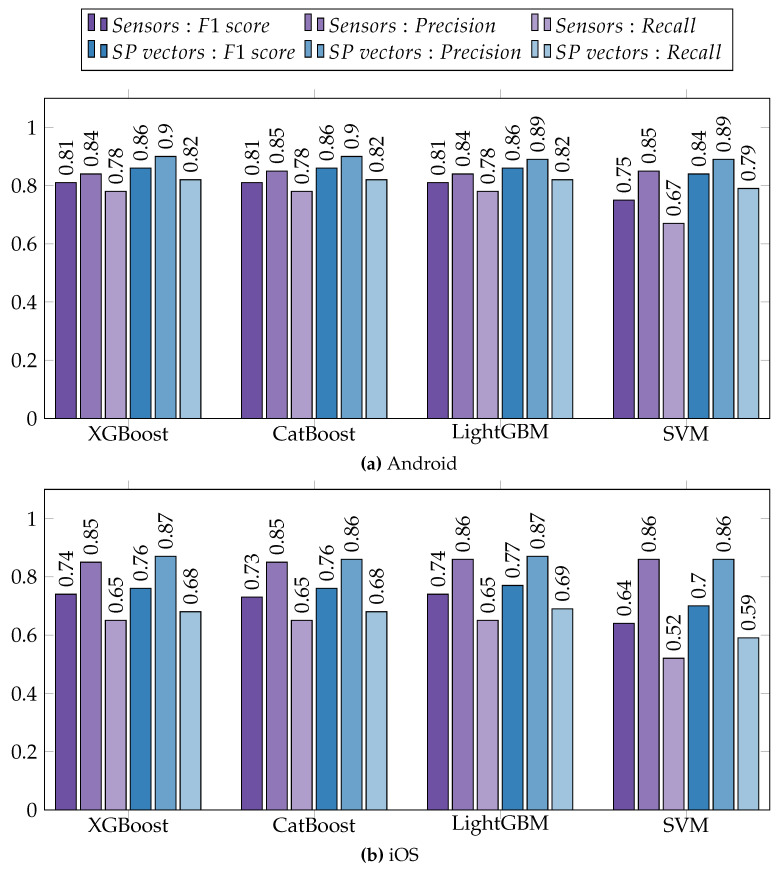
Prediction of depression severity using sensor data and symptom profile vectors (SP vectors) as input. (**a**) Results from the Android dataset and (**b**) results from the iOS dataset.

The results are depicted in Figure 5, which is divided into separate subfigures for Android (Figure 5a) and iOS (Figure 5b) datasets due to differences in the collected sensor sets. The bars colored in purple represent the results obtained by utilizing extracted sensor features as input for the machine learning models. Conversely, the bars colored in blue indicate the results obtained when utilizing the symptom profile vectors as input.

For both datasets, utilizing symptom profiles as input yielded higher $F_{1}$ scores compared to employing sensor data, and this trend was consistent across all the tested models. On average, there was an improvement in the $F_{1}$ score by 0.06 for the Android dataset and an improvement of 0.04 for the iOS dataset. Recall increased by 0.06 on average for Android and by 0.04 for iOS, whereas precision experienced an increase of 0.05 for Android and a modest increase of 0.01 for iOS. It is worth noting that most of the achieved results were lower for iOS compared to Android. This discrepancy may be attributed to the smaller dataset size and other potential factors discussed in Section 6.1.

Additionally, we investigated the impact of manually adjusting the weights of symptom profile vector elements corresponding to specific depressive symptoms. The reasoning behind this experiment is outlined in Section 5.2. To assess the predictive performance, we used techniques specific to each classifier. For the XGBoost classifier, we employed the feature_weights parameter in the fit() function, while in the case of LightGBM and CatBoost classifiers, we utilized the feature_contri and feature_weights parameters respectively, during initialization. In the case of the SVM classifier, we used a scaling factor of two for the symptom profile vector elements associated with the symptoms of depressed mood and diminished interest. The results of this experiment exhibited a marginal increase by less than 0.01 in both $F_{1}$ score and precision, while recall demonstrated minimal change for both Android and iOS datasets. Based on these findings, we concluded that artificially doubling the weights of specific symptom elements did not significantly influence the performance of the model. However, exploring alternative weight adjustment factors and different combinations of symptoms for weight calibration could serve as a future research direction.

### 6.3. Symptom Profiling and Depression Severity

**Figure 6:**
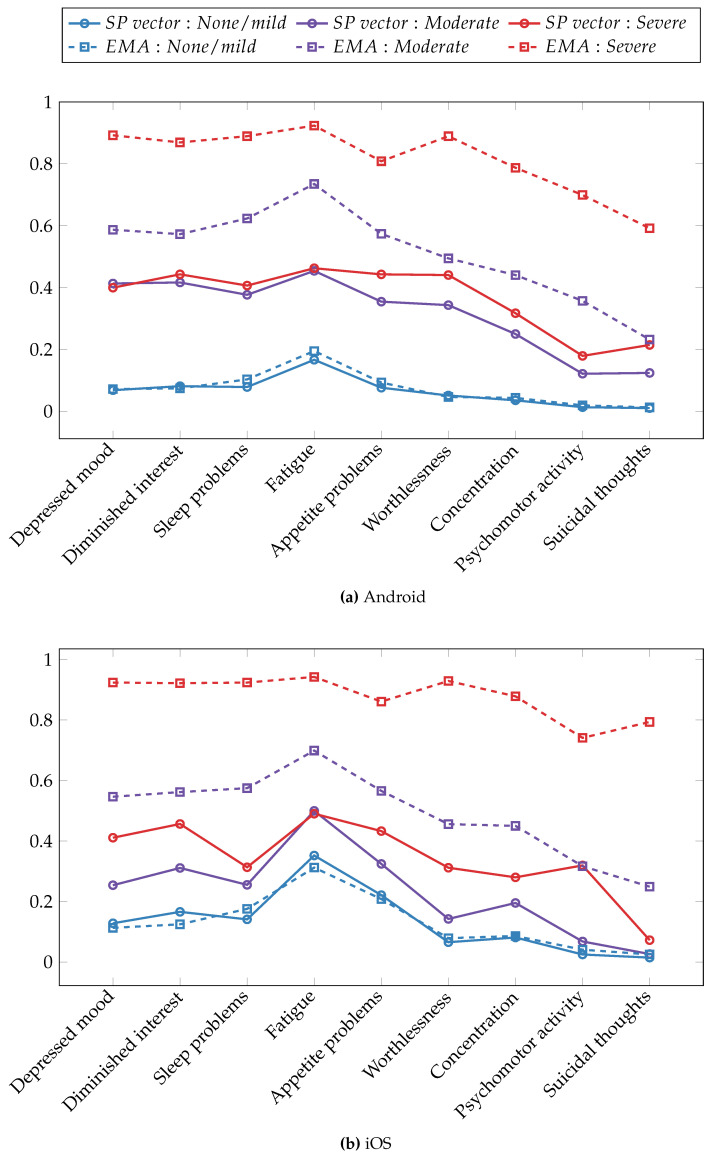
Averaged symptom profile vectors and rescaled PHQ-9 item scores across depression severity groups. (**a**) The Android dataset and (**b**) the iOS dataset.

To assess the effectiveness of the symptom profile metric in categorizing participants into distinct depression severity groups, we computed the average values of the symptom profile vector elements for each of the three depression levels. Subsequently, we compared these averages with the normalized scores obtained from the original self-report responses, which range from 0 to 1. The comparison of symptom profile values with original self-report scores offers a perspective on the precision and reliability of our approach in capturing the complexity of depressive symptomatology. Figure 6 illustrates the average symptom profile values alongside the corresponding original ground truth answers for each depressive symptom separately for Android (Figure 6a) and iOS (Figure 6b) datasets. In the figure, solid lines represent the values derived from the symptom profile metric, while dashed lines depict the original scores obtained through EMAs. The color scheme employs blue, purple, and red to indicate not/mildly depressed, moderately depressed, and severely depressed participants, respectively.

Regarding the Android dataset, the symptom profile vector was most effective for binary classification in terms of the separability of depression severity groups. The not/mildly depressed group exhibited a distinct separation from the moderately depressed and severely depressed groups. When distinguishing between the moderately and severely depressed groups, the symptom profile scores were most effective in differentiating problems with appetite, feeling of worthlessness, and suicidal thoughts symptoms. In terms of the dominant depressive symptoms across depression severity levels, the fatigue symptom demonstrated a higher magnitude compared to other depressive symptoms within the not/mildly and moderately depressed groups. This was supported by both the symptom-profiling and original EMA responses. Within the severely depressed group, multiple symptoms exhibited similarly high magnitudes when assessing both symptom profile values and original EMA scores. The symptom profiling performed most accurately when estimating original EMA scores for the not/mildly depressed group, but its estimation for the moderate and severe groups was less precise.

Turning to the iOS dataset, the symptom profile metric performed less effectively at separating depression groups compared to the Android dataset. This could be attributed to several factors, including a smaller number of participants, fewer available data sources, and a larger amount of missing data due to operating system restrictions. Nevertheless, the symptom profile vectors still managed to separate the three depression severity groups. The most significant differences between symptom profile values for not/mildly and moderately depressed groups were achieved for symptoms related to depressed mood, diminished interest, and fatigue. When distinguishing between the moderately and severely depressed groups, the symptom profile vector was most effective in capturing the difference in the psychomotor activity symptom. Similar to the Android dataset results, the most commonly experienced symptom across non-depressed individuals and those with mild to moderate depression was fatigue, as demonstrated by both EMA item scores and the symptom profile vectors. The strength of depressive symptoms was underestimated by the symptom profiles for depressed groups, while it provided fairly accurate estimations for the not/mildly depressed group.

## 7. Discussion and Future Work

The present study aimed to assess the effectiveness of machine learning in predicting both depression severity and related symptoms from passive data. Predicting specific depressive symptoms is crucial because relying solely on the total score of the PHQ-9 to predict depression misses the diverse phenotypes of depression and may hinder achieving a high predictive accuracy rate. To address this, we introduced a novel approach called symptom profiling, which demonstrated its potential as a valuable tool for predicting and categorizing depression severity using smartphone data while providing an overview of the specific depressive symptoms that an individual is experiencing. In the initial phase of our data analysis, we employed machine learning models to predict the presence or absence of depressive symptoms. The results highlighted the robust predictive capabilities of our approach, particularly for cognitive symptoms such as diminished interest, depressed mood, feelings of worthlessness, and concentration difficulties. Regarding the classification of depression severity, we found that using symptom profile vectors as inputs for machine learning models consistently outperformed using sensor data in terms of classification accuracy. Our proposed approach demonstrates that digital phenotyping can be utilized not only for predicting depression but also for monitoring depression severity and the dynamics of specific depressive symptoms.

While our study provides valuable insights, we acknowledge certain limitations and suggest areas for potential improvement in future digital phenotyping research. One notable limitation of our dataset is its ground truth imbalance, a common issue encountered in digital phenotyping research due to the low frequency of depression in the general population [47]. The discrepancy between the symptom profile metric and the original PHQ-9 self-reports increases with depression severity. One potential explanation is the imbalanced dataset, given that we have the fewest number of participants in the severely depressed group. Future research should prioritize the recruitment of more severely depressed participants and consider incorporating clinical interviews in participant selection, if feasible. Additionally, despite our efforts to include participants from different age groups, the majority of our sample consists of young adults below the age of 30. To enhance the generalizability of our proposed methods, it is essential to validate our findings on a sample of individuals encompassing a broader age range, including older adults.

Regarding the collected data, we rely on PHQ-9 questionnaires as self-report measures, which consist of one question per depressive symptom, amounting to nine questions in total. However, augmenting the data collection with Beck Depression Inventory (BDI-II) or Quick Inventory of Depressive Symptomatology (QIDS) [48] alongside PHQ-9 may offer a more comprehensive understanding of depressive symptoms. Both BDI-II and QIDS offer more detailed and fine-grained questions for certain symptoms. For instance, while the PHQ-9’s question related to sleep problems inquires about general trouble sleeping, the QIDS questionnaire expands this query into four sub-questions: (1) time taken to fall asleep, (2) quality of sleep during the night, (3) instances of waking up too early, and (4) excessive sleeping. Given that the questionnaires are widely utilized in clinical settings for detecting depression, combining them could provide more comprehensive data for analysis.

In terms of sensor data, augmenting the features with additional voice-related parameters (e.g., speaking frequency) could yield valuable insights into the social aspect of depression and potentially enhance classification accuracy. Furthermore, sleep duration features are powerful predictors of depression [23]. Calculating sleep duration from sensor data can be achieved using the method proposed by [49], or alternatively, special APIs [50,51] for Android and iOS operating systems respectively. Finally, to improve the practicality of mobile applications for depression monitoring, extending passive data-collection intervals is beneficial in reducing battery consumption.

## 8. Conclusions

This research builds upon prior studies, indicating that digital phenotyping employing passive smartphone sensor data could serve as a tool for capturing short-term fluctuations in depression. This paper takes one step further in investigating the viability of utilizing smartphone data for the prediction of depressive symptoms and depression severity. A non-clinical cohort of 381 participants was involved in this study, confirming the feasibility of the depression-severity-classification model using the widely recognized PHQ-9 assessment as a ground truth. Additionally, we explored methods to enhance depression-severity-prediction accuracy and proposed a novel method involving symptom profiling, which represents experienced depressive symptoms. We conducted a comparison of machine learning performance for depression severity prediction, evaluating the utilization of sensor data directly against the use of symptom profile vectors as input. The proposed approach with symptom profiles consistently demonstrated improved accuracy across both Android and iOS datasets.

## Figures and Tables

**Table 1:** Literature review: mental health with passive sensing.

Author (Year)	Sample Size and Type	Study Length	EMA	Pre/Post Test	Device
Wang et al. (2014) [18]	48 college students	10 weeks	stress, sleep, activity, mood, social, exercise, behaviour	PHQ-9, flourishing scale, PSS, UCLA	Android
Ben-Zeev et al. (2015) [19]	47 young adults	10 weeks	stress rating	PHQ-9, PSS, UCLA	Android
Canzian et al. (2015) [7]	28 adults	71 days on average	PHQ-8	-	Android
Saeb et al. (2015) [8]	40 adults	2 weeks	-	PHQ-9 (pre-test only)	Android
Saeb et al. (2016) [20]	48 college students	10 weeks	-	PHQ-9	Android
Boukhechba et al. (2018) [21]	72 college students	2 weeks	positive, negative mood rating	SIAS, DASS, PANAS	Android
Wang et al. (2018) [22]	winter term: 56 college students, spring term: 27 college students	winter term: 9 weeks, spring term: 9 weeks	PHQ-4	PHQ-8	Android, IOS, wearable
Xu et al. (2019) [23]	phase I: 188 college students, phase II: 267 college students	phase I: 106 days, phase II: 113 days	-	BDI-II	Android, wearable
Narziev et al. (2020) [6]	20 college students	4 weeks	5-item depression survey	PHQ-9, BDI-II, STAI	Android, wearable
Razavi et al. (2020) [24]	412 adults	14 days	-	BDI-II	Android
Ware et al. (2020) [12]	phaseI: 79 college students, phase II: 103 college students	phase I: October 2015–May 2016, phase II: February 2017–Decemebr 2017	phase I: PHQ-9, phase II: QIDS	PHQ-9 and QIDS (pre-test only)	Android, iOS
Opoku Asare et al. (2021) [25]	629 adults	22 days on average	PHQ-8	-	Android
Ross et al. (2023) [26]	295 adults	-	PHQ-8	-	iOS

**Table 2:** Data sources and their properties.

Data Source	Description	Android	iOS	Optional
Applications usage	application category and duration of usage	event-based	-	no
Calendar	total number of calendar events	4 h	4 h	no
Call log	number and duration of incoming, outgoing, missed calls	event-based	event-based	no
Camera	cropped face images	event-based	event-based	yes
GPS	latitude and longitude	15 min	15 min	no
Gravity	magnitude of gravity in x, y and z directions	15 min	15 min	no
Keystroke log	number of key presses, backspaces, auto-correction, typing duration, and number of unique applications	event-based	-	no
Light	illuminance in lux	15 min	-	no
Microphone	sound energy and pitch	15 min	event-based	yes
Music	song title and artist name	event-based	-	no
Notifications	notification event, click-through rate, and decision time	event-based	-	no
Pedometer	number of steps	event-based	event-based	no
Physical activity	duration of still, walking, running, cycling, being in vehicle activities	event-based	event-based	no
Screen	duration of unlocked screen state	event-based	event-based	no
Significant motion	number of events when sudden motion occurs	event-based	event-based	no
Social Networking Service (SNS)	social media metrics (e.g., number of followers and posts)	24 h	-	yes
Stored media	number of images stored	4 h	4 h	yes
Short Messaging Service (SMS)	number of characters in incoming text messages	event-based	-	no
Wi-Fi	number of nearby access points	30 min	-	no

**Table 3:** Extracted features.

Data Source	Feature Name	Device OS
Additional	day of the week, gender, age group	Android, iOS
Applications usage	social/finance/media/tools/work/study/lifestyle/significant/non-significant apps mean usage duration and variance [39]	Android
Call log	number of missed/incoming/outgoing calls, incoming/outgoing calls mean duration and variance	Android, iOS
GPS	time spent at home/work/other places, total travelled distance, maximum distance from home, number of visited places, standard deviation of displacement [8]	Android, iOS
Gravity	mean and variance of gravity magnitude in x, y, z directions	Android, iOS
Keystroke log	number of key presses, number of backspace presses, number of unique apps, number of auto corrections, mean duration and variance of typing in significant/non-significant apps	Android
Light	mean and variance of light intensity	Android
Microphone	mean and variance of sound loudness, number of times pitch is detected	Android, iOS
Notifications	number of arrived/clicked notifications, number of unique apps, mean and variance of decision time	Android
Pedometer	number of steps	Android, iOS
Physical activity	mean and variance of being active (walking, running, cycling)/inactive (still, in vehicle)	Android, iOS
Screen	mean and variance of unlocked screen state	Android, iOS
Stored media	mean number of image files stored	Android, iOS
Wi-Fi	mean number of access points	Android

**Table 4:** Prediction of individual depressive symptoms.

Device OS	Depressive Symptom	F1 Score	Precision	Recall
Android	Diminished interest	0.80	0.85	0.75
	Depressed mood	0.80	0.85	0.75
	Sleep problems	0.77	0.84	0.70
	Fatigue	0.69	0.80	0.61
	Appetite problems	0.77	0.84	0.70
	Felling of worthlessness	0.83	0.86	0.81
iOS	Diminished interest	0.68	0.79	0.59
	Depressed mood	0.70	0.80	0.62
	Sleep problems	0.66	0.79	0.57
	Fatigue	0.64	0.77	0.55
	Appetite problems	0.63	0.75	0.54
	Feeling of worthlessness	0.75	0.82	0.70
	Concentration problems	0.76	0.83	0.71

## Data Availability

The data presented in this study are available on request from the corresponding author. The data are not publicly available due to privacy restrictions.
